# Current Hospital Topics

**Published:** 1907-04-13

**Authors:** 


### CURRENT HOSPITAL TOPICS.

### A Hospital Manager's Handbook.

We are often asked, by members of committees and governors of hospitals, for a book containing in a handy form most of the material facts about the various types of hospitals, with which it is desirable that every hospital manager should make himself familiar. The new volume of "Burdett's Hospitals and Charities" 1907, recently published, now in its eighteenth year, contains most things of importance in connection with each hospital and kindred institution, situated not only in the United Kingdom and the British Empire, but also in the United States of America. The preliminary chapters, which deal with current hospital matters and contain a mass of information of essential value to the economy committees especially of every hospital, have more than usual interest this year. Amongst hospital managers none hold a more important position than the active members of the honorary medical staff. They, as individuals, are supposed to know most about hospitals, and "Burdett's Hospitals and Charities" in its present form is a handy book which most active practitioners habitually use. The chapter on Hospitals in the United States of America, by Dr. Goldwater, is of special interest to medical and lay managers of British hospitals, as its perusal will convince our correspondents and readers. A hundred and one points of detail which arise, in connection with the administration of hospitals and medical institutions, may for the most part be readily answered by a reference to the particulars given in this book, about each type or institution concerning which precise information is desired. There are some very striking statistics of great interest to the medical profession, as well as to the public and hospital managers generally in the first chapter devoted to the misuse of hospitals. We share the Editor's confidence that the time is approaching for the institution of some well thought out and uniform plan, to limit the present number of free patients receiving relief at the voluntary hospitals in this country.

### The Radcliffe Infirmary, Oxford.

After a useful existence of 136 years, this institution may be said to be in a sound position financially. The report just issued shows, however, that the committee are in the habit of bringing forward each year the debt of the previous year. The tendency during the last five years has been to slightly reduce the amount of this debt, which stood at £2,676 in 1902, and was apparently reduced to £1,166 by the end of 1906, though the actual deficit for that year was £304. We would venture to point out to the committee, seeing that they must have had to pay interest all these years on the debt brought forward, that it is not good business to continue this practice when the invested funds exceed £54,800. Had the committee sold £2,000 stock five years ago and wiped off the whole debt, they would have been able to save money, and would certainly have shown financial acumen. The average annual expenditure is under £10,000 a year, and any voluntary hospital which has invested funds equal to five years' expenditure, possesses an ample reserve for all contingencies. No voluntary hospital needs a greater margin of invested funds than the equivalent of five years' expenditure, for such an institution's dependence upon the good-will and support of the people of each generation to whose needs it ministers, is one of the essentials which secures the highest efficiency in administration for voluntary hospitals. We are glad to note that the accounts are now kept upon the uniform system, and that the ladies are actively interesting themselves in the support of the Radcliffe Infirmary. It is gratifying to find, too, that the financial position is such that Captain Rynd should have small difficulty in securing an ample revenue to meet the out-goings of each year, providing the committee decide, as we hope they will do, to realise stock, and wipe out the balance of old debt which has too long cumbered the annual statements of revenue and expenditure. We are glad to know that the League of Mercy contributed £110 to this institution during 1906. This is a new source of revenue, which would never have been obtained had it not been for the initiative of the Prince of Wales, the Princess Victoria, and the presidents, vice- presidents, and members of the League of Mercy.

